# Noninvasive automatic detection of Alzheimer's disease from spontaneous speech: a review

**DOI:** 10.3389/fnagi.2023.1224723

**Published:** 2023-08-24

**Authors:** Xiaoke Qi, Qing Zhou, Jian Dong, Wei Bao

**Affiliations:** ^1^School of Information Management for Law, China University of Political Science and Law, Beijing, China; ^2^AI Speech Co., Ltd., Suzhou, China; ^3^Information Technology Research Center, China Electronics Standardization Institute, Beijing, China

**Keywords:** Alzheimer's disease, spontaneous speech, dataset, machine learning, deep learning, classification, optimization

## Abstract

Alzheimer's disease (AD) is considered as one of the leading causes of death among people over the age of 70 that is characterized by memory degradation and language impairment. Due to language dysfunction observed in individuals with AD patients, the speech-based methods offer non-invasive, convenient, and cost-effective solutions for the automatic detection of AD. This paper systematically reviews the technologies to detect the onset of AD from spontaneous speech, including data collection, feature extraction and classification. First the paper formulates the task of automatic detection of AD and describes the process of data collection. Then, feature extractors from speech data and transcripts are reviewed, which mainly contains acoustic features from speech and linguistic features from text. Especially, general handcrafted features and deep embedding features are organized from different modalities. Additionally, this paper summarizes optimization strategies for AD detection systems. Finally, the paper addresses challenges related to data size, model explainability, reliability and multimodality fusion, and discusses potential research directions based on these challenges.

## 1. Introduction

Alzheimer's disease (AD) is one of the most prevalent neurological disorders. It primarily affects older adults, with age being a significant risk factor for its development. Recently, AD has become one of the main causes of death among people over 70 years old (Alzheimer's Association, 2019). The World Health Organization (WHO) has reported that dementia currently affects over 50 million people worldwide, with millions of new diagnoses each year (World Health Organisation, 2020), likely increasing to above 152 million in 2050 (Nichols et al., 2022). According to Alzheimer's Society (2020), the prevalence of AD is also expected to increase, as indicated by the doubling of AD cases in individuals over the age of 60 approximately every 4-5 years. Among individuals over the age of 80, the likelihood of developing AD is estimated to be one in three (Ritchie and Lovestone, 2002). AD is characterized by a continuous deterioration of cognitive and functional abilities in individuals over time, encompassing domains such as language, memory, attention and executive function (Nestor et al., 2004; American Psychiatric Association, DSM-5 Task Force, 2013). Therapeutic interventions have shown the greatest efficacy before neuronal degeneration occurs in the brain (Nestor et al., 2004). Therefore, early identification of these deficits is crucial, as it has the potential to significantly impede the progression of cognitive impairments and enable the preservation of cognitive functions in patients (Dubois et al., 2009).

To date, there has been a lot of research focused on developing methods for detecting AD, including neuropsychological tests [e.g., self-report questionnaires, the mini-mental state examination (MMSE) (Folstein et al., 1975)], and neuroimaging techniques [e.g., magnetic resonance imaging (MRI) (Jack et al., 2008), positron emission tomography (PET) (Samper-González et al., 2018)]. Although these methods can offer relatively accurate diagnoses of AD, they suffer from some drawbacks. Neuroimaging and cerebrospinal fluid analysis are expensive, time-consuming, invasive, and require validation by neurologists and manually clinical settings. Cognitive assessments and self-report questionnaires are tedious and may not have good test-retest reliability and validity. Therefore, there is a need for more practical and reliable methods for AD detection that are less invasive and can be used in a natural environment.

On the contrary, speech-based methods have the potential to provide non-invasive, effective, simple, and inexpensive tools for automatically detecting AD. There are several reasons why speech is so useful for this purpose. First, speech is closely related to cognitive status, and it has been widely used as the main input in various mental health assessment applications. The most significant correlation with AD is the difference in speech comprehension, reasoning, language production, and memory functions, which can result in a reduction in vocabulary and verbal fluency, as well as difficulties in performing daily tasks related to semantic information (Forbes-McKay and Venneri, 2005). Hoffmann et al. (2010) compared four temporal parameters in individuals with AD and control subjects, namely articulation rate, speech tempo, hesitation ratio and rate of grammatical errors. Significant differences were observed between the two groups, with hesitation ratio showing particularly notable disparities. These findings indicate that temporal aspects of speech play a vital role in the differentiation of AD from other neurodegenerative disorders and can even aid in the detection of early-stage AD. Additionally, the studies focusing on the speech of individuals with AD have consistently demonstrated that their acoustic and linguistic abilities are significantly impacted, even during the early stages of the disease, leading to noticeable differences when compared to individuals without AD (Ahmed et al., 2013; Szatloczki et al., 2015). These distinctive differences observed between individuals with AD and those without AD can be harnessed for the purpose of detecting AD through speech analysis. Second, spontaneous speech can be easily accessed anywhere, as it only requires a device with a recording function. Speech can also be used as a cost-effective long-term monitoring approach.

Motivated by these, research has increasingly focused on utilizing spontaneous speech to extract information for the automatic detection of AD. The studies can be broadly categorized into two main directions: extracting discriminative features from speech data to identify AD patients, and designing effective classification models to achieve high detection performance. In the feature domain, spontaneous speech of AD patients exhibits many distinguishable characteristics, such as lower speech rate, more frequent and longer hesitations, obscurer pronunciation, and longer pauses, compared to non-AD (NAD) participants (Hoffmann et al., 2010; Szatloczki et al., 2015). These distinctions can be leveraged to extract linguistic and acoustic features for the automatic detection of AD. Linguistic features encompass the linguistic content and structure of speech and can be extracted from manually annotated transcripts or generated through automatic speech recognition (ASR) systems. These features include measures of parts-of-speech (POS) tags (Bucks et al., 2000), grammatical constituents (Fraser et al., 2014), lexical diversity (Fraser et al., 2016a), global vectors (GLoVe) (Pennington et al., 2014), word2vec (Mirheidari et al., 2018), and deep embeddings using techniques such as bidirectional encoder representations from transformers (BERT) (Yuan et al., 2020) and other neural network methods (Pan et al., 2019). Acoustic features refer to the characteristics of speech that are related to its physical properties, and can be extracted using traditional handcrafted or deep embedding techniques, such as Fourier analysis, Mel-frequency cepstral coefficients (MFCCs) (Alhanai et al., 2017), term frequency-inverse document frequency (TF-IDF) (Ramos et al., 2003), and wav2vec (Baevski et al., 2020). Besides, other features can also provide useful information for AD detection, including speaker-specific attributes such as age, gender, and interactional features (e.g., turn-taking patterns).

In the model domain, the models for AD detection from speech can be divided into three types based on different modal input. Speech-based models are built with acoustic features as model input, and text-based models exploit linguistic information as model input. Multimodal-based models combine features from speech and text modalities as model input. These models are trained mainly based on statistical machine learning such as linear discriminant analysis (LDA), decision tree (DT), support vector machine (SVM) and random forests (RF), and deep learning (DL) algorithms, including fully connected neural network (FCNN), convolutional neural network (CNN), recurrent neural network (RNN), long short-term memory (LSTM) network, gated recurrent unit (GRU), and Transformer-based models.

However, automatic detection of AD is still a challenging task from spontaneous speech. One reason lies in the lack of specialist data due to the challenges associated with collecting a large amount of transcribed speech recorded from AD patients and the limited availability of clinical professionals. Then, another reason is that many NNs appear black boxes, making it challenging to understand the underlying features driving their predictions and give meaningful interpretations.

The paper presents a review of automatic detection systems for from spontaneous speech. The main contributions can be summarized as follows:

- We conduct a comprehensive review and summary of the development of each module in AD detection systems, focusing on the data collection module, feature extraction module and classification module. This provides a comprehensive understanding of the various components involved. Notably, our paper focuses on the advancements made for AD detection technologies especially in the last three years, providing an up-to-date analysis of the state-of-the-art. This distinguishes our work from previous review publications such as Petti et al. (2020) covering the period between 2013 and 2019, Pulido et al. (2020) covering 2005–2018, de la Fuente Garcia et al. (2020) covering 2000–2019, (Vigo et al., 2022) covering 1996-2020, and (Mart́ınez-Nicolás et al., 2021) covering 2010-2020.
- Following a handbook-style approach, we provide a detailed description of the features and classifiers usually used in AD detection models. This allows readers to easily access information on AD detection without the need to search through numerous papers.
- We compile a summary of the state-of-the-art performance on popular datasets from recent papers, providing insights into the corresponding technologies used for feature extraction, classifiers, and optimization strategies.
- We provide a discussion of the existing challenges in AD detection, with a focus on practical applications aspects such as data, modality, explainability and reliability. Additionally, we propose potential future directions to address these challenges.

The paper starts with a description of the task of automated AD detection from spontaneous speech (Section 2). Then, some recent public datasets are introduced and features extracted from speech and text are detailed shown in Section 3. In Section 4, we review popular classification algorithms used in AD detection and discuss strategies for improving performance. Section 5 presents a discussion of the challenges that still need to be addressed. Finally, Section 6 provides conclusions and outlines potential ideas for future work.

## 2. Task description

AD is thought to be the most prevalent neurodegenerative condition with common signs of memory and cognitive decline. AD detection and treatment is greatly helpful for delaying irreversible brain damage, and thus important in AD research. Since a key marker of early AD is decline in speech and language functionality, like the reduction of vocabulary and verbal fluency, this allows us to extract information from speech or/and the corresponding transcripts to distinguish AD and non-AD (NAD). Therefore, the automatic AD detection task is to determine a category c^*^ between AD and NAD with a higher probability given data **d**, which is formulated as


(1) $$\begin{array}{l} c^{*}=\underset{c=\left\{AD,NAD\right\}}{\text{max}}p\left(c|\text{d}\right). \end{array}$$

### 2.1. System architecture

**Figure 1:**
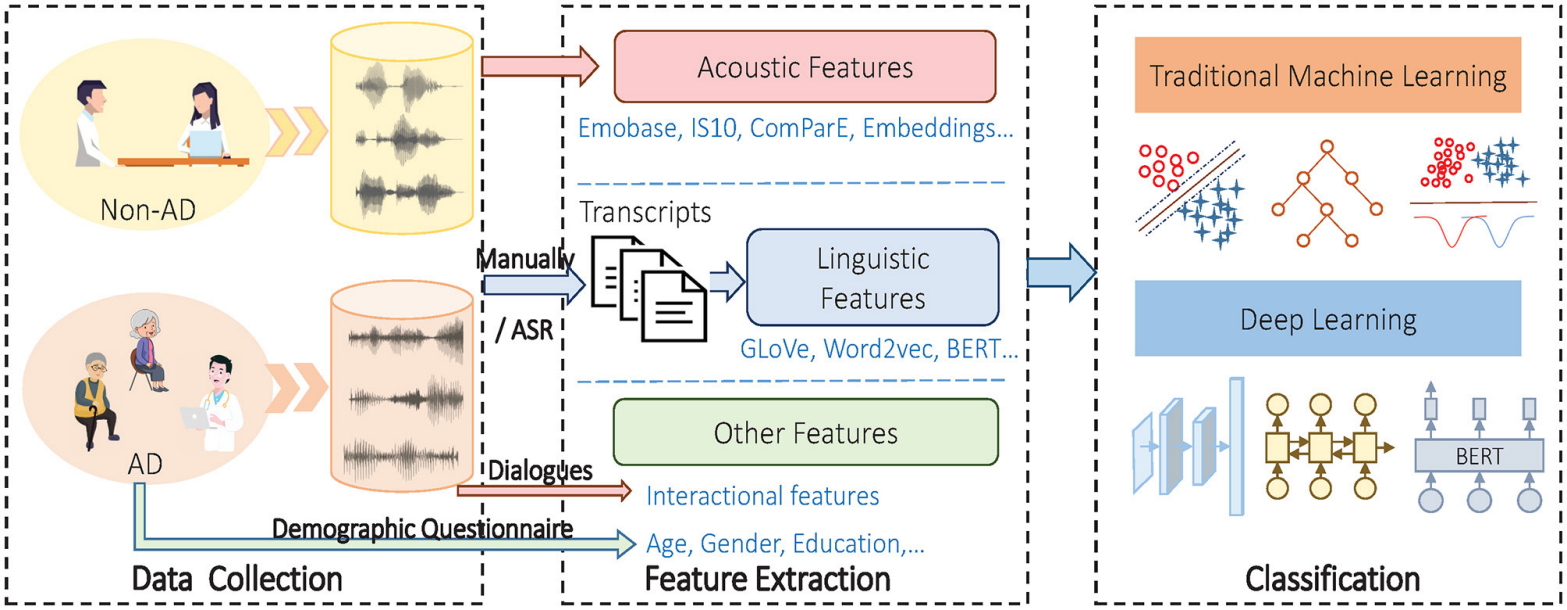
A hierarchy of automatic detection of AD from spontaneous speech.

To solve the problem, a typical system architecture is demonstrated in Figure 1. The process of automation detection of AD can be categorized into three stages: data collection, feature extraction and classification.

First, the data **d** are collected by recording speech from both individuals with and without AD using various methods. After data collection, it is common to partition the dataset into a training set, a validation set, and a test set. The training set is used to train the model, while the validation set is used for fine-tuning and hyperparameter tuning. Finally, the test set is kept separate and used for unbiased evaluation of the trained classifier. Given that the original audio waves and transcripts include both valuable and redundant information for AD detection, it becomes essential to extract relevant features, emphasizing the informative aspects. The process can be conceptualized as mapping the raw data **d** to meaningful representations **F** that capture the relevant characteristics for AD detection, expressed as


(2) $$\begin{array}{l} \text{F}=f\left(\text{d}\right). \end{array}$$

The core is to extract discriminate features to classify AD and NAD as accurately as possible, which should be designed carefully. Three types of features are generally exploited for this purpose. One is acoustic features extracted from speech data. Many acoustic features such as MFCC, wav2vec2.0 are related to the severity of AD. Another linguistic features are obtained from transcripts which are usually from manual annotation or an ASR system, containing GLoVe, word2vec, BERT embedding and so on. Then, there are some other features including individual attributes such as age and gender, and interactional features from dialogues. More detailed description about feature extraction will be found in Section 3.2. Therefore, instead of Equation 1, the practice uses the features to detect AD, which is expressed as


(3) $$\begin{array}{l} c^{*}=\underset{c=\left\{AD,NAD\right\}}{\text{max}}p\left(c|f\left(\text{d}\right)\right)=\underset{c=\left\{AD,NAD\right\}}{\text{max}}p\left(c|\text{F}\right). \end{array}$$

A classification model is used to address the issue of Equation 3. The modeling methods contain two categories: traditional statistical machine learning algorithms and DL algorithms. Statistical machine learning algorithms usually have clear theories and reduction process and thus have having desirable interpretability, such as LDA, DT, SVM and RF. On the other hand, DL algorithms have been proven to achieve a better performance in many fields, such as CNN, RNN, LSTM and Tranformer-based models. Several canonical classification models will be introduced in detail in Section 4.

### 2.2. Evaluation metrics

The system for AD classification is typically evaluated by metrics including the accuracy (*A*), precision (*P*), recall (*R*) and *F*_1_ score, which are defined as


(4) $$\begin{array}{l} A=\frac{TN+TP}{TN+TP+FN+FP}, \end{array}$$

(5) $$\begin{array}{l} P=\frac{TP}{TP+FP}, \end{array}$$

(6) $$\begin{array}{l} R=\frac{TP}{TP+FN}, \end{array}$$

(7) $$\begin{array}{l} F_{1}=\frac{2PR}{P+R}, \end{array}$$

where *TP* represents the number of true positives, *TN* represents the number of true negatives, *FP* denotes the number of false positives and *FN* is false negatives.

### 2.3. Study selection process

To comprehensively review the aforementioned systems, we conducted a search for relevant articles published within the current year. First, our primary focus is on the automatic detection of AD based on speech data. Therefore, our inclusion criteria are to select articles that employ speech and/or text analysis and ML methods for the automatic detection of AD. On the other hand, we excluded studies related to other dementia conditions, such as Parkinson's disease, as well as those utilizing non-speech data like MRI. Additionally, studies solely relying on traditional statistical analysis for AD detection without incorporating ML methods were also excluded. By applying these specific criteria, we aim to narrow our focus to research that utilizes ML-driven approaches for automatic AD detection using speech data.

Then, to obtain the relevant articles, we conducted a thorough literature search using prominent academic databases like Google Scholar and conference proceedings, with a particular emphasis on conferences like Interspeech and ICASSP, renowned for their focus on speech processing and provide valuable contributions to the field of automatic AD detection through speech analysis. To refine our search and target relevant articles, we employed inclusion criteria and exclusion criteria. We applied specific inclusion and exclusion criteria to refine our search and target relevant articles. Initially, we used keywords related to “Alzheimer's disease” OR “AD” OR “dementia” AND “speech” to retrieve articles. Subsequently, we manually selected or excluded articles after careful reading to ensure their relevance to our research focus. Notably, the focus of this review is on automated AD detection from speech patterns using ML-based systems. While Mini Mental State Examination (MMSE) scores are commonly used as a quantitative measure of cognitive impairment and provide valuable insights into the disease's dynamics in monitoring the progression of AD, the vast and continuously evolving literature on AD progression and MMSE prediction goes beyond the scope of this review. Moreover, many studies employed a similar architecture for MMSE prediction (Rohanian et al., 2021; Jin et al., 2023; Tamm et al., 2023), which results in significant overlap with AD detection in terms of features and ML techniques. Due to space limitations and to maintain a clear focus on AD detection from speech data, specific aspects related to MMSE prediction were not explored in this review.

Furthermore, to ensure the most up-to-date information, we primarily searched for papers published within the last 3 years, aiming to capture the latest advancements and developments in the field of automatic AD detection.

By combining these search strategies, we gathered a robust collection of relevant studies, enriching our literature review with comprehensive insights and valuable findings related to the automatic detection of AD from speech data.

## 3. Materials

### 3.1. Datasets

A dataset used for automatic AD detection from speech is obtained by recruiting participants with and without AD and collecting recordings from them using various methods, including neuropsychological tests and natural conversations. Neuropsychological tests include but not limited to the following tests.

- The picture description test (Croisile et al., 1996; Forbes-McKay and Venneri, 2005). The picture description test involves presenting a subject with an picture and requesting them to provide a detailed description of the depicted scenario within a specified time frame.
- Verbal fluency test: animal category (Hart et al., 1988; Randolph et al., 1993). During verbal fluency assessment, participants are given a specific category, typically related to animals (e.g., dog, cat, fish), and are instructed to generate as many different words as possible within a time limit.
- Boston naming test (BNT) (Koss et al., 1996). BNT has been predominantly used to assess naming ability for the degree of language disturbances in clinical neuropsychology. A typical form consists of 60 pictures ordered from easy to difficult, and the subjects are requested to name them (Kaplan et al., 2001).
- Logical memory test (Greene et al., 1996; Rabin et al., 2009). Logical memory test is especially useful for detecting relatively mild retrieval problems, which includes word list learning, delayed recall, recognition and constructional praxis (Rosen et al., 1984). During these selected tests, spontaneous speech data will be recorded. Some of them are then manually transcribed.

Several public datasets are published for automatic detection of AD from spontaneous speech, which allows researchers to easily access the study of AD detection. Table 1 presents a compilation of public datasets, including their respective dataset names, reference papers, spoken languages, modalities, and participant information. These datasets were selected by following the criteria of public availability, and widespread usage in experiments for automatic AD detection.

**Table 1:** This table shows a summary of datasets for AD detection.

**Dataset**	**References**	**Language**	**Modality**	**Source**
DementiaBank	Boller and Becker, 2005	English, German, Mandarin, Spanish, Taiwanese	Audio, Video, or Text	310 AD patients, 241 HCs
Pitt	Becker et al., 1994	English	Audio, Text	208 AD patients, 104 HCs, 85 unknown diagnosis
Lu	MacWhinney et al., 2011	Chinese	Audio, Text	52 AD patients in Mandarin and 16 AD in Taiwanese
Ivanova	Ivanova et al., 2022	Spanish	Audio, Text	74 AD patients, 197 HCs, 90 MCI
WLS	Herd et al., 2014	English	Audio	10,317 participants
CCC	Pope and Davis, 2011	English	Audio, Text	125 AD patients, 125 non-AD controls
Chile	Sanz et al., 2022	Spanish	Audio, Text	21 AD patients, 18 Parkinson's disease patients, and 16 HCs
ILSE	Martin et al., 2000	German	Audio, Text (part)	Over 8,000 hours of recorded speech data from more than 1,000 participants over a long period of 20 years. 5.4 % AD patients, 5.4% MCI, 60.8% HCs in the third measurements
ADReSS	Luz et al., 2020	English	Audio, Text	78 AD patients, 78 HCs
ADReSSo	Luz et al., 2021	English	Audio	87 AD patients, 78 HCs
NCMMSC2021	Competition Group, 2021	Mandarin	Audio, Text	26 AD patients, 44 HCs, 54 MCI
ADReSS-M	Luz et al., 2023	English, Greek	Audio	148 AD patients, 143 HCs

Note that the datasets Pitt, Lu, Ivanova and WLS are subsets of DementiaBank. ADReSS and ADReSSo are subsets of Pitt, which have been acoustically enhanced and reorganized.

**DementiaBank** (Boller and Becker, 2005) is the largest publicly available database, which is a multilingual data bank consisting of 15 datasets in English, German, Mandarin, Spanish and Taiwanese. DementiaBank contains 241 narrations from individuals without any cognitive impairment (referred to as healthy controls or HCs) and 310 narrations from those diagnosed with dementia. These narrations were collected annually from 1983 to 1988 from participants aged between 45 and 90 years. They were asked to perform various tasks, such as the picture description test. Audio recordings with/without textual transcriptions, annotated at the utterance level and synchronized with the audio, are available for each case in the dataset. After that, more data will be added to DementiaBank. *Pitt* corpus (Becker et al., 1994) is a widely used subset of DementiaBank. Pitt were gathered longitudinally from 104 elderly controls, 208 with probable and possible AD, and 85 unknown diagnosis participants. Responses to four language tasks were recorded, including one task of Cookie Theft picture description for all participants, and three tasks of verbal fluency, sentence construction and story recall for AD group only. *Lu* corpus from DementiaBank comprises interview recordings of 52 AD patients in Mandarin and 16 AD patients in Taiwanese, by performing tasks such as the Cookie theft picture description, category fluency, and picture naming (MacWhinney et al., 2011). Ivanova et al. (2022) collected recordings from a total of 361 Spanish native speakers aged over 60, including 74 AD patients, 197 HCs and 90 individuals with MCI. They were asked to read the first paragraph of the novel “The Ingenious Gentlemen Don Quixote of La Mancha.” The **Wisconsin Longitudinal Study (WLS)** is a long-term research project that aims to understand the life course and the factors influencing individuals' lives. It includes a random sample of 10,317 Wisconsin high school graduates surveyed over nearly 60 years from 1957 to 2011 (Herd et al., 2014). While the WLS does not currently provide dementia-related diagnoses in its metadata, it offers valuable data on demographics, socioeconomic status, health behaviors, and cognitive abilities, making it a relevant resource for AD research.

**The Carolinas Conversation Collection (CCC) dataset** (Pope and Davis, 2011) is a collection of transcribed speech and video of conversations with people over the age of 65. It consists of over 200 consented conversations with 125 subjects who have one or more of 12 chronic conditions and over 400 conversations with 125 AD patients, recorded at least twice a year. These conversations cover topics related to the participants' daily lives and health issues and are conducted with interviewers.

**The Chile dataset** (Sanz et al., 2022) was created from 55 native Spanish speakers, including 21 AD patients, 18 Parkinson's disease (PD) patients, and 16 HCs. The participants were asked to perform seven language tasks covering different communicative behaviors, such as describing daily routine and primary interests, recounting a pleasant memory as well as an unpleasant memory, describing a modified picnic scene and a picture depicting a family working in an unsafe kitchen, and immediately recalling and narrating a one-minute silent animated film. Through these tasks, linguistic patterns express diverse and partly predictable. The audio was recorded using laptops in a quiet room, and the transcripts were generated using ASR and then manually revised.

**The Interdisciplinary Longitudinal Study on Adult Development and Aging (ILSE)** (Martin et al., 2000) was collected with the aim of studying the challenges posed by rapidly aging societies in both East and West Germany. It consists of more than 8,000 hours of recorded speech over a long period of 20 years from 1,000+ individuals diagnosed with AD, cognitive decline, mild cognitive disorder, vascular dementia, as well as HCs. Each participant was asked to complete up to four measurements and provide detailed responses to open-ended questions. So far, 380 hours of ILSE were manually transcribed (Weiner et al., 2016a).

**ADReSS (The Alzheimer's Dementia Recognition through Spontaneous Speech)**, derived from the Cookie session of Pitt, is a “balanced and acoustically enhanced” challenge dataset hosted by Interspeech2020 conference (Luz et al., 2020). ADReSS contains the recordings of 78 AD patients and 78 HCs with a matched age and gender. The data from Pitt were enhanced with noise removal, and then segmented using voice activity detection. After volume normalization, over 5000 speech segments were generated.

**ADReSSo (The Alzheimer's Dementia Recognition through Spontaneous Speech only)** is a dataset used in Interspeech2021 Challenge (Luz et al., 2021). Two tasks were designed to record speech of participants: a semantic fluency task and a Cookie Theft picture description task. The resulting training set contained 166 instances with 87 AD patients and 79 HCs. There were also other 71 instances with 35 AD patients and 36 HCs in the test set. No transcripts are provided with ADreSSo.

**NCMMSC's (National Conference on Man-Machine Speech Communication) AD dataset** (Competition Group, 2021) is used for NCMMSC2021 AD Recognition Challenge. The recordings were collected from a total of 124 Chinese speakers, containing 26 AD patients, 44 HCs and 54 MCIs. They were required to complete tasks including picture description, fluency test and free conversation with the interviews. The resulting dataset contained 280 samples with the duration of each sample in about 30-60 seconds.

**ADReSS-M (Multilingual Alzheimer's Dementia Recognition through Spontaneous Speech)** is an ICASSP 2023 Signal Processing Grand Challenge that aims to explore the extraction of universal acoustic features from speech data to facilitate multilingual detection of AD (Luz et al., 2023). The ADReSS-M dataset consists of audio recordings of picture descriptions obtained from 148 AD patients and 143 HCs, in English and Greek languages. The dataset is divided into three splits: an English training split, a Greek sample split, and a Greek test split. The English training set was collected from 122 AD patients and 115 HCs. Participants were asked to describe the Cookie Theft picture in English during the recording session. On the other hand, the Greek sample split and test split consist of spontaneous speech descriptions of a different picture in the Greek language. The sample split includes recordings from 8 subjects, with 4 AD patients and 4 HCs, while the test split involves data from 46 participants, with 22 AD patients and 24 HCs. It is noteworthy that the ADReSS-M dataset's splits were meticulously balanced for both age and gender.

### 3.2. Feature extraction

After a dataset is prepared, it is necessary to extract features from spontaneous speech before classification. Feature extraction is expected to separate the relevant features for AD detection from redundant and irrelevant data. After that, feature selection or/and feature fusion is implemented to improve the detection performance by selecting a subset of more discriminative representative features and fusing them. As shown in Figure 1, three types of features can be extracted: acoustic features from audio, linguistic features from the transcripts and other features.

#### 3.2.1. Acoustic features

Acoustic features may change in individuals with AD due to the physiological and cognitive changes associated with the disease. Firstly, AD can impact the coordination and control of the muscles involved in speech production, including the articulatory and vocal folds muscles. This can result in changes in articulation, such as imprecise consonant production, reduced vocal range, and alterations in speech rhythm. These changes can be reflected in features like MFCCs, which capture spectral information, and measures like jitter and shimmer, which assess perturbations in fundamental frequency and amplitude. Secondly, AD is characterized by progressive cognitive decline, including impairments in memory, attention, language, and executive functions. These changes can affect speech production, leading to alterations in acoustic features. For example, individuals with AD may exhibit difficulties in word retrieval, sentence construction, and maintaining coherent speech, which can be reflected in changes in speech rate, pauses, and speech fluency. Then, individuals with AD may experience changes in vocal quality, including hoarseness, breathiness, and reduced vocal intensity. These changes can be detected by jitter, shimmer, and harmonics-to-noise ratio, which provide measures of vocal stability, roughness, and clarity. Additionally, language impairments, such as word-finding difficulties, semantic deficits, and syntactic errors, are commonly associated with AD. These can influence the structure and content of speech, leading to changes in acoustic features related to language, such as pauses, speech rate, and the distribution of acoustic energy across different frequency bands.

Based on the recent papers, acoustic features used in AD detection can be divided into frame-level features, embedding features and paralinguistic features including prosody, disfluency and emotional features.

##### 3.2.1.1. Frame-level features

Frame-level acoustic features are directly derived from audio files. The time and frequency characteristics and statistical functionals are captured, such as MFCCs, *F*_0_ and energy distribution. Frame-level features can be easily obtained by public audio processing toolkits, such as OpenSMILE (Eyben et al., 2010) and Kaldi (Povey et al., 2011). From these toolkits, different acoustic feature sets can be extracted from the raw audio files as follows.

- Emobase (Schuller et al., 2010). It includes a range of audio features including MFCC, *F*_0_, *F*_0_ envelope, line spectral pairs (LSP) and intensity features, along with their first and second-order derivatives.
- IS10 (Eyben et al., 2013). The set includes MFCC, loudness, *F*_0_ envelope, LSP, voicing probability, jitter local, jitter derived perturbation parameter, and shimmer local features.
- AVEC (Valstar et al., 2013). The AVEC feature set comprises various energy, spectral, and voicing-related features, along with their statistical properties, regression features, and functionals related to local minima and maxima.
- ComParE (Schuller et al., 2013). The ComParE feature set includes a comprehensive collection of acoustic features that capture various aspects of speech and non-speech signals. Some specific features within the ComParE set include "logarithmic harmonic-to-noise ratio, voice quality features, Viterbi smoothing for *F*_0_, spectral harmonicity and psychoacoustic spectral sharpness" (Schuller et al., 2013). Finally, statistical functionals are calculated to summarize the distributional properties of these features.
- eGeMAPS (Eyben et al., 2015). The feature set attempts to reduce the number of other sets to 88 features with theoretical significance, and thus detect physiological changes in voice production. These features encompass MFCC, loudness, spectral flux, jitter, shimmer, *F*_0_, *F*_1_, *F*_2_, *F*_3_, alpha ratio, Hammarberg index, slope *V*_0_, and their statistical functionals.
- Bag-of-Audio-Words (BoAW) (Schmitt and Schuller, 2017). BoAW contains the quantization of acoustic low-level descriptors (LLDs), including MFCC, log-Mel, and the ComParE features.
- Multi-resolution Cochleagram features (MRCGs) (Chen et al., 2014). MRCGs are generated by mimicing the human auditory filters. Firstly, the audio signal is passed through a gammatone filter and then decomposed in the frequency domain using multiple levels of resolution. The low-resolution level encodes spectrotemporal information, while the high-resolution level focuses on capturing local information. By combining these different levels of resolution, a time-frequency representation is obtained to effectively capture the multi-resolution power distribution of the audio signal.

##### 3.2.1.2. Acoustic embeddings features

Embedding features are generated from the embedding layer based on deep neural network.

- VGGish (Hershey et al., 2017). VGGish is an acoustic embedding model which is pretrained using a CNN-based structure on YouTube's Audio dataset. VGGish extracts and transforms the audio into high-level feature vectors.
- Speaker Embeddings. Speaker embeddings aim to extract information related to speaker identity in a compact form. The typical speaker embeddings contains i-vectors (Dehak et al., 2010) and x-vetctors (Snyder et al., 2018). I-vector embeddings are extracted based on a Universal Background Model (UBM) and a Gaussian Mixture Model (GMM) to model the variability of the speaker and channel. X-vectors are a type of speaker representation and extracted using deep neural networks. These embeddings contain information related to gender, emotion, and articulatory, phonatory and prosodic information. Pérez-Toro et al. (2021) extracted x-vectors based on a trained Time delay neural network for AD detection.
- Neural network. Popular deep neural network architectures, such as DNN, CNN, can also generate embedding features by selecting the output of a specific layer. These embeddings capture higher-level representations of the input data learned by the neural network. Cummins et al. (2020) investigated Siamese network combined with contrastive loss functions and end-to-end convolutional neural network (CNN), and found that these systems can capture the features related to different production mechanisms and extract the characteristic of AD speech from all. Pan et al. (2020) proposed Sinc-CLA as a feature extractor for the classification of neurodegenerative disorders, mild cognitive impairment and healthy controls.
- Wav2vec2.0 (Baevski et al., 2020). Wav2vec2.0 is a self-supervised end-to-end ASR system developed by Facebook AI Research. Wav2vec2.0 contains a multi-layer convolutional feature encoder which encodes raw wave into latent representations, a quantization module for masking and a Transformer to get textualized representationsoptimized by minimizing a connectionist temporal classification (CTC) loss. Since Wav2vec2.0 can also capture the speaker and language characteristics in the audio (Fan et al., 2020), the outputs of transformer layers can be extracted as the embedding representations of the input utterances. Pan et al. (2021) used the last hidden state of Wav2vec2.0 as acoustic embedding features.

##### 3.2.1.3. Prosody

Prosody defines patterns of intonation and stress, which is easily affected by cognitive impairments. Prosodic measures focus on temporal aspects, intensity, voice quality, interruptions, voice periods, and variation in *F*_0_, as well as statistical functionals.

##### 3.2.1.4. Disfluency

AD patients often experience difficulties with language and cognitive skills. As the disease progresses, they may exhibit slower speech rate, longer pauses or breaks between words or sentences, and increased difficulty in finding the right words, resulting in disfluencies in their speech. There are different types of disfluency features to show the ability of subjects in organizing language, such as percentage of broken words, repetitions, sound prolongations, self-repairs (Shriberg, 1994) and pauses. Pauses include filled pauses and unfilled pauses. Cmmon filled pauses contain “uh,” “um,” “oh,” “well,” laughter, and so on. Yuan et al. (2020) calculated word frequencies and showed that AD patients had potential to use more 'uh', laughter and meaningless words like “well,” “oh,” but less “um,” compared to HCs. Moreover, the durations of unfilled pauses calculated from forced alignment were analyzed and the results showed that AD patients had more and longer pauses. As a result, the durations can be extracted for distinguishing AD patients as pause features.

##### 3.2.1.5. Emotional embeddings

AD patients often experience a reduced ability to perceive and express emotions due to their memory loss (Henry et al., 2009), and thus emotional features can be extracted to capture relevant information about the emotional state of AD patients. A continuous emotion state can be expressed by a three-dimensional vector with valence, arousal, and dominance. Pérez-Toro et al. (2021) trained three models to respectively obtain three factors by combining CNN and GRU, and extracted the output of the embedding layer as emotional features.

#### 3.2.2. Linguistic features

Linguistic features undergo changes in individuals with AD due to the progressive nature of the condition, which affects various cognitive and language-related processes. AD is characterized by language impairments, and as the disease advances, individuals may encounter difficulties in word retrieval, comprehension of complex grammatical structures, construction of grammatically correct sentences, and maintenance of coherent discourse. These language impairments are evident in alterations in vocabulary usage, sentence structure, and overall linguistic fluency. Word-finding challenges may lead to frequent pauses and the substitution of words with similar-sounding alternatives, consequently impacting the flow and coherence of speech. Furthermore, AD can result in decreased verbal expression abilities, including reduced output, shorter and less complex sentences, and a decrease in the overall quantity of speech. As a result, the range of vocabulary becomes limited, and the utilization of syntactic structures may diminish. Additionally, AD can affect the organization and coherence of discourse, leading to unrelated responses, difficulties in maintaining topic coherence, and challenges in adhering to conversational conventions. Pragmatic impairments may also arise, encompassing difficulties in appropriate language usage within social contexts. These challenges can involve struggles with turn-taking, adherence to conversational norms, and comprehension of non-literal language, such as sarcasm or metaphors.

Linguistic features used in AD detection encompass various aspects such as syntax, semantics, word embeddings, sentence embeddings, and more. These features can also be categorized as traditional handcrafted features and deep embeddings.

##### 3.2.2.1. Traditional features

Traditional handcrafted features derived from theories of Linguistics, which include features related to syntactic, semantic, and lexical diversity. Specifically, it includes the following features.

- Parts-of-speech (POS). The production of different POS reflects language changes, including a decrease in the number of nouns, and an increase in the number of pronouns, adjectives and verbs (Bucks et al., 2000). POS and related statistical features comprise the frequency of different POS occurrences, dependency tags in the subject's transcript, ratios of nouns to verbs, pronouns to nouns, and more.
- Syntactic complexity. The syntactic complexity of the picture descriptions can be assessed through various measures, including the mean length of utterances, T-units (Hunt, 1970), clauses, the height of the parse tree and the statistics of Yngve depth (Yngve, 1960).
- Grammatical constituents. A set of context-free grammar features derived from the parse tree analysis has shown the potential to differentiate between individuals with agrammatic aphasia and HCs during a story-telling task (Fraser et al., 2014). These features includes the frequency of different grammatical constituents, as well as the rate, proportion and average length of different phrases (e.g., noun phrases, verb phrases and prepositional phrases).
- Vocabulary richness or lexical diversity. It can be measured by unique word count, type-token ratio (TTR), moving-average type-token ratio, Brunét's index and Honoré's statistic (Fraser et al., 2016a). TTR denotes the ratio of the total number of unique words to the overall text length, which is sensitive to text length, while the other three measures provide an unbiased metric of lexical richness without being influenced by text length.
- Repetitive and diverse features. AD disorder impacts memory, resulting in AD patients potentially using a more repetitive and less diverse vocabulary compared to HCs (Nicholas et al., 1985; Syed et al., 2021). To quantify it, some features are extracted such as TTR, the number of repetitive words, and the number of sweepback caused by self-corrections. A bag-of-words measures the cosine distance between each pair of utterances, with a result of zero to indicate the two identical utterances.
- TF-IDF (Ramos et al., 2003). TF-IDF is used to determining a word's relative importance in a specific document compared to its overall frequency across the entire document corpus. Common words in a single document tend to achieve a higher score than those like articles and prepositions. Given the documents **D** = {*d*_1_, *d*_2_, *d*_3_, ...}, where *d*_*i*_ denote a document in the corpus, the TF-IDF of a word *w* in a document *d*_*i*_ can be calculated by Salton and Buckley (1988)
  (8) $$\begin{array}{l} T_{w}^{d_{i}}=c_{w}^{d_{i}}\log\frac{\left|\text{d}\right|}{c_{w}^{\text{d}}}, \end{array}$$
  where $c_{w}^{d_{i}}$ denotes the number of times the word *w* appears in the document *d*_*i*_. |**D**| represents the total number of documents in the corpus. $c_{w}^{\text{d}}$ denotes the number of documents in which the word *w* appears.

##### 3.2.2.2. Deep embeddings

- Word2Vec. Word2Vec represents a class of neural network models, such as skip-gram and the continuous bag-of-words (CBOW). Word2Vec can encode semantic information from unlabeled data by producing embedding vectors. These vectors can be used for the semantic similarity and many other NLP tasks. The procedure for CBOW as an example is to train a NN using neighbor words to predict a target word. Specifically, text segment is first represented using the average of normalized word embeddings such as one-hot encodings, and the results are fed to a RF classifier (Bojanowski et al., 2017). Word vectors are obtained from the activations of a hidden layer.
- BERT-based embeddings.
  BERT is a powerful unsupervised and deep pretrained model (Kenton and Toutanova, 2019). By utilizing the encoder part of the Transformer architecture, BERT transforms words/sentences in a corpus into embedding feature vectors, which can be further used for classification. BERT has spawned various variants. One widely used variant called RoBERTa (Robustly Optimized BERT approach) (Liu et al., 2019) has been developed and gained significant attention. RoBERTa benefits from the larger training corpus and optimized training procedure to learn more robust representations and exhibit improved performance across multiple tasks. Wang et al. (2022b) used fine-tuned text embedding networks, such as BERT and Roberta, to extract linguistic information, and then used majority voting to fuse the decisions.

##### 3.2.2.3. Readability features

Considering that AD patients show difficulties in understanding the meaning of complex words and syntax (Croisile et al., 1996), readability features are extracted for AD detection to capture the complexity of language, such as gunning fog index (GFI) (Gunning, 1969), automated readability index (ARI) (Smith and Senter, 1967), the simple measure of Gobbledygook (SMOG) grading (Mc Laughlin, 1969) and the ratio of unique words. GFI and ARI are designed to evaluate the number of years of formal education required for a person to comprehend a text on the first reading, which are calculated as Martinc and Pollak (2020)


(9) $$\begin{array}{l} GFI=\frac{0.4\left(N_{w}+100N_{lw}\right)}{N_{s}}, \end{array}$$

(10) $$\begin{array}{l} ARI=\frac{4.71N_{c}}{N_{w}}+\frac{0.5N_{w}}{N_{s}}-21.43, \end{array}$$

where *N*_*c*_, *N*_*w*_ and *N*_*s*_ denote the number of characters, words and sentences, respectively. *N*_*lw*_ is the number of long words longer than 7 characters. SMOG grading is used to assess the reading level and comprehension difficulty of health messages, expressed as


(11) $$\begin{array}{l} SMOG=3.1291+1.0430\sqrt{30N_{syl}/N_{s}}, \end{array}$$

where *N*_*syl*_ is the number of polysyllabic words in samples of 30 sentences.

##### 3.2.2.4. Acoustic and linguistic feature fusion

Besides separate acoustic and linguistic features, there are techniques providing a way of fusing acoustic and linguistic features. For example, Haider et al. (2019) developed an active data representation (ADR) to fuse bi-modal features at a word and sentence level, which can model temporal aspects of text and speech. The ADR features include cluster counts, cross-modality word embeddings, pause, centroid embeddings, embedding velocity and centroid velocity, duration (Haider et al., 2019; Martinc et al., 2021). Martinc et al. (2021) combined ADR with TF-IDF weighted bag-of-n-grams to model semantics better.

##### 3.2.2.5. Other features

Other features encompass various aspects relevant to AD detection, such as age and gender obtained from a demographic questionnaire or natural conversations during the recording process, and interactional features from dialogues.

##### 3.2.2.6. Meta features

Meta-features, such as age, gender, education, genetic factors and so on, are demographic or clinical characteristics of individuals that are not directly related to the disease but can have a significant impact on its development, progression, and presentation. The relationship between AD and meta-features has been a subject of significant research interest in the field of neurodegenerative diseases. For example, aging is associated with various changes in the brain, including the accumulation of amyloid plaques and neurofibrillary tangles, which are hallmark features of AD pathology. Andersen et al. (1999) has shown that gender may play a role in AD susceptibility. Women tend to have a higher risk of developing AD compared to men. Education level has been associated with cognitive reserve, which refers to the brain's ability to adapt and function despite damage. Higher education levels have been linked to greater cognitive reserve, potentially delaying the onset of cognitive decline and AD symptoms.

##### 3.2.2.7. Interactional features

During dialogue conversations, temporal and interactional aspects are distinctive between AD patients and the interviewers. For example, the subjects with AD are older people with longer lapse and lower speech rates compared to the interviewers within the conversation. Thus, an interactional feature set can be extracted to quantify the interactions between patients and interviewers for AD detection. Nasreen et al. (2021b) exploited 32 features to describe the interaction within the natural conversations, including speech rate (measured in syllables per minute), turn length (measured in words per turn), floor control ratio (indicating the proportion of speech time by AD patients relative to the total conversation duration), normalized total duration of short and long pauses (the total duration of pauses normalized by the total duration without pauses), and so on.

Based on the available studies, it is evident that a wide range of features have been extracted with the primary aim of obtaining more discriminative features for effective AD detection. Furthermore, there is a noticeable trend in the studies toward transitioning from handcrafted features to utilizing deep embedding representations. This transition highlights the growing interest in leveraging advanced techniques to capture higher-level representations for AD detection.

## 4. Methods

After learning features from bi-modal speech and text data, they are used to build a classification model for recognizing AD patients. There are two typical types of algorithms for this end: statistical machine learning methods and deep learning methods.

### 4.1. Statistical machine learning

#### 4.1.1. Support vector machine (SVM)

SVM (Cortes and Vapnik, 1995) is a popular type of supervised learning algorithm used for classification and regression tasks. SVM aims to find a hyperplane that separates the data points into different classes by maximizing the margin between the classes, i.e., the distance between the closest data points from each class to the hyperplane. The data points that are closest to the hyperplane are called support vectors, and used to define the hyperplane. Moreover, SVM can map the input data points into a higher-dimensional space using a kernel function, and then different classes may be more easily recognized. Zargarbashi and Babaali (2019) extracted acoustic representations of I-vectors and D-vectors for speech and N-gram representations for transcription text, and used SVM on these features to recognize AD, achieving a classification accuracy of 83.6% using the Pitts Corpus. Wang et al. (2022b) selected classifiers from five classification models: SVM, LDA, Gaussian process (GP), multilayer perceptron (MLP), and extreme gradient boost (XGB). The experimental results showed that SVM classifier combined with BERT and Roberta features achieved best performance among all.

#### 4.1.2. Logistic regression

Logistic regression (LaValley, 2008) is used to analyze and model binary or categorical outcomes. The model first uses the logistic function to compute the probability of the binary outcome, and then utilizes the predictors to estimate the coefficients of the logistic function, which determines the relationship between the predictors and the probability of the binary outcome. Liu et al. (2020) used a logistic regression model trained on spectrogram features extracted from speech data for recognizing AD. Shah et al. (2021) tested the performance of SVM, LR and majority vote classifiers when using acoustic features only, linguistic features only and the combined features, and showed that an ensemble of acoustic-based and language-based models yielded the best performance.

#### 4.1.3. Linear discriminant analysis (LDA)

LDA (Balakrishnama and Ganapathiraju, 1998) aims to find a linear combination of features that maximizes the separation between different classes while minimizing the variance within each class. The core concept of LDA is to project the original high-dimensional data onto a lower-dimensional subspace that retains the most discriminatory information. This subspace is defined by the eigenvectors of the between-class scatter matrix and is referred to as the discriminant subspace. Weiner et al. (2016b) developed a LDA model for classification and achieved a classification accuracy of 85.7%.

#### 4.1.4. k-Nearest neighbors (KNN)

KNN (Fix, 1985) identifies the k-nearest neighbors to a given data point based on a distance metric, and then uses the majority vote of these neighbors to classify the data point or estimate the value of the target variable. One of the advantages of KNN is its simplicity and interpretability, as the decision boundary is determined by the data itself. However, KNN can be computationally expensive for large datasets and may suffer from the curse of dimensionality.

#### 4.1.5. Decision tree (DT)

A decision tree is a tree-like model that consists of a series of decisions and their possible consequences (Quinlan, 1986). Each internal node of the tree represents a decision based on the value of a feature, and each leaf node represents a class or a value of the target variable. DT is popular due to its interpretability, flexibility, and ease of implementation. Mirzaei et al. (2018) used three classification models: KNN, SVM and DT to classify AD, MCI and HCs.

#### 4.1.6. Random forest (RF)

RF (Breiman, 2001) is an ensemble learning method that combines multiple decision trees to improve the accuracy and robustness of the model. Each tree in the forest is trained on a subset of the data, and the final prediction is made by taking the majority vote of all the trees. RF is known for its high accuracy, scalability, and resistance to overfitting. Hernández-Doḿınguez et al. (2018) trained SVM and RF to distinguish between HCs and MCI, and the results provide insights into the effectiveness of SVM and RF classifiers in the early diagnosis of MCI. In Edwards et al. (2020), the effectiveness of multiscale (word and phoneme level) features was explored using five different classification models: LDA, KNN, DT, RF and SVM, achieving a maximum classification accuracy of 79.2%.

### 4.2. Deep learning

#### 4.2.1. Convolutional neural network (CNN)

CNN (LeCun et al., 1998) is composed of multiple convolutional layers that learn a hierarchy of features from the input data, followed by one or more fully connected layers that perform the classification task. CNN is known for its ability to automatically learn spatial and temporal features from the data, and has been widely studied and applied in various fields, such as self-driving cars, medical image analysis, and robotics. Warnita et al. (2018) utilized a gated CNN and achieved an accuracy of 73.6% for AD detection on the Pitt corpus.

#### 4.2.2. Recurrent neural network (RNN)

RNN (Werbos, 1988) is composed of a network of recurrently connected nodes that allow to maintain a state or memory of previous inputs. RNN can handle variable-length input sequences and commonly used for sequence modeling tasks. However, RNN suffers from gradients exploding or vanishing during training. To overcome this issue, long short-term memory (LSTM) is designed to use a memory cell and several gating mechanisms to selectively retain or forget information from previous inputs, which allows the network to preserve a long-term memory of past inputs. Koo et al. (2020) used an improved convolutional RNN to identify AD. Pan et al. (2019) exploited a bidirectional hierarchical RNN with an attention layer for AD detection. Ablimit et al. (2022) used CNN-GRU-Attention and FCNN to process features and make model fusion. Yang et al. (2022) constructed AD detection model using two LSTM layers after the convolutional layers.

#### 4.2.3. Transformer models and variations

The Transformer (Vaswani et al., 2017) is a groundbreaking deep learning model architecture that introduced the attention mechanism and revolutionized the processing of sequential data. Unlike RNNs that rely on sequential processing, the Transformer enables parallelization and more efficient training. The Transformer model consists of two key components: the encoder which processes the input sequence and extracts its contextual information, and the decoder which generates the output sequence. The key innovation of the Transformer lies in its attention mechanism, which allows the model to focus on different parts of the input sequence while processing each word or token. It helps the model capture long-range dependencies and contextual information effectively.

BERT is based on the Transformer architecture developed by Google (Kenton and Toutanova, 2019). BERT is pretrained on large amounts of unlabeled text data to predict missing words in a sentence by considering the context of both the left and right surrounding words. This bidirectional approach enables BERT to capture deeper contextual relationships and produce more meaningful representations of words. After pretraining, BERT can be fine-tuned on specific NLP tasks by adding task-specific layers, and the entire model is fine-tuned on labeled task-specific data. Fine-tuning allows BERT to adapt its representations to the specific requirements of the target task. BERT has been used for AD detection by fine-tuning it on a dataset of speech samples from individuals with and without AD (Balagopalan et al., 2021). ERNIE (Enhanced Representation through Knowledge Integration) is a language representation model based on the Transformer architecture (Zhang et al., 2019). ERNIE is designed to capture rich semantic representations of text by incorporating techniques such as knowledge masking, sentence-level discourse representation, and knowledge graph.

### 4.3. Optimization and performance

Based on the above classical learning methods, more research is focusing on finding optimization techniques to improve the performance of automatic AD detection. To achieve this goal, the studies have focused on two main aspects: extracting more distinguishing features, and building more powerful classification models to detect AD.

Table 2 summarized performance comparison of AD detection on different datasets when using different optimization methods, in terms of the average accuracy *A*(%), precision *P*(%), recall *R*(%) and *F*_1_(%). Only the most notable studies chosen to show in Table 2, to provide a comprehensive understanding of the current state-of-the-art in AD detection. When diving into how to achieve better results, the typical optimization methods can be categorized as follows in detail.

**Table 2:** This table shows a performance comparison of AD detection on different datasets in terms of the average accuracy *A*(%), precision *P*(%), recall *R*(%) and *F*_1_(%) defined in Equation 4.

**Dataset**	**References**	**Modality**	**Feature**	**Classifier**	***A***	***P***	***R***	***F*_1_**	**Optimization**
Pitt	Wang et al., 2022a	Text (ASR)	BERT, RoBERTa	SVM	91.7	88.5	95.8	92.0	ASR improvement, Model fusion
	Sarawgi et al., 2020	Speech, Text	Disfluency, ComParE, Interventions	MLP	88.0	92.0	82.0	88.0	Prosody features, Model fusion
	Ye et al., 2021	Text (ASR)	BERT	SVM	88.0	82.0	96.0	88.0	ASR improvement
ADReSS	Wang et al., 2022b	Text (ASR)	BERT, RoBERTa	SVM	93.8	92.0	95.8	93.9	Model fusion
	Martinc et al., 2021	Speech, Text	ADR, Bag-of-n-gram	k-means clustering, RF	93.8	-	-	-	ADR features, Model fusion
	Wang et al., 2022b	Text (Manual)	BERT, RoBERTa	SVM	91.7	91.7	91.7	91.7	Model fusion
	Martinc et al., 2021	Text	Bag-of-n-gram	k-means clustering, RF	89.6	-	-	-	-
	Yuan et al., 2020	Text	Pauses	ERNIE	89.6	90.2	89.5	89.6	Task-specific features
	Syed et al., 2020	Text	BERT, RoBERTa, DistilBERT	SVM, LR	85.4	-	-	-	Model fusion
	Sarawgi et al., 2020	Speech, Text	Disfluency, ComParE, Interventions	MLP	83.0	83.0	83.0	83.0	More features, Model fusion
ADReSSo	Pan et al., 2021	Text (ASR)	ASR hypotheses, Confidence score	BERT	84.5	84.7	84.6	84.5	ASR features
	Syed et al., 2021	Text (ASR)	BERT	LR	84.5	-	-	84.5	Model fusion
	Rohanian et al., 2021	Speech, Text (ASR)	Acoustic, GloVe, Disfluency, Pause	LSTM with gating	84.0	-	-	-	Prosody features
	Zhu et al., 2021	Speech, Text (ASR)	Wav2vec, Pause	BERT	83.1	83.6	83.0	83.0	Pause features
	Qiao et al., 2021	Text (ASR)	Complexity, Disfluency	LR, ERNIE, BERT	83.1	83.5	83.0	83.0	Model fusion
	Wang et al., 2021	Speech, Text (ASR)	X-vector, Linguistic	CNN + attention	80.3	81.9	80.1	81.0	Model fusion
	Pan et al., 2021	Speech	Wav2vec	RF	74.7	75.0	74.6	74.5	-
CCC	Nasreen et al., 2021a	Speech	Acoustic, Interactional	SVM, LR	90.0	90.5	90.0	89.5	Interactional features
ADReSS-M	Jin et al., 2023	Speech	Acoustic, Disfluency	Swin transformer, RF	86.7	-	-	-	Model fusion
	Tamm et al., 2023	Speech	eGeMAPS	attention pooling+MLP	82.6	88.9	-	80.0	Fine tuning
	Mei et al., 2023	Speech	Low-pass filtered speech	Wav2vec2	73.9	-	-	-	Fine tuning
	Shah et al., 2023	Speech, Text (ASR)	Duration, Pause, Confidence score, Meta	LR	69.6	-	-	-	Feature combination
	Chen et al., 2023	Speech	IS10	SVM	69.6	69.2	75.0	72.0	Paralinguistic features

#### 4.3.1. Extraction of discriminative features

Features play a crucial role in determining the performance of a classifier. Numerous studies have made efforts to extract features by analyzing the impact of Alzheimer's disease (AD) on patients, focusing on characteristics that distinguish them from individuals without AD, include longer pauses, increased disfluency, slower response during dialogues, and more. These discriminative features include pauses (Yuan et al., 2020; Rohanian et al., 2021; Zhu et al., 2021), disfluency (Sarawgi et al., 2020; Qiao et al., 2021; Rohanian et al., 2021), interactional features (Nasreen et al., 2021a), cognition features (Sarawgi et al., 2020), ADR features (Martinc et al., 2021). By identifying and incorporating such specific features into the classification process, researchers aim to enhance the accuracy and effectiveness of AD detection methods. For instance, Nasreen et al. (2021a) obtained promising results using interactional features alone with an accuracy of 87%. Yuan et al. (2020) encoded pauses into three bins: long (over 2 s), medium (0.5-2 second) and short (under 0.5 second), and reported 89.58% accuracy when combining with ERNIE. Rohanian et al. (2021) extracted features (disflency, pauses, and language model probabilities) and achieved an accuracy of 84% with a classifier of LSTM with gating. Zhu et al. (2021) introduced non-semantic information, i.e., sentence-level pauses based on wav2vec, and BERT classifier achieved an accuracy of 83.1%. Pan et al. (2021) adopted ASR for feature extraction and BERT for classification, and finally achieved 74.65% and 84.51% accuracy for the acoustic-only and best linguistic-only features, respectively. Paralinguistic features, such as duration, pauses, and others, have been shown to be effective for multilingual AD detection. Shah et al. (2023) extracted paralinguistic features, including word-level duration, pause rate, as well as meta-features and confidence scores of each word from the ASR model, for cross-lingual AD detection. Chen et al. (2023) utilized paralinguistic features for cross-lingual AD detection and achieved excellent results when compared to pre-trained features. Therefore, incorporating more discriminative features has the potential to increase the accuracy of AD detection.

#### 4.3.2. Model fusion

Model fusion can further improve the classification performance by combining data from multiple models. Two types of fusion methods are usually used, feature fusion and decision fusion. Feature fusion refers to the process of combining features from different sources or modalities at the input stage of a model. For feature fusion, different features can be concatenated, weighted, or combined in other ways to form a more comprehensive or informative representation of features. On the other hand, decision fusion combines the outputs or decisions of multiple models at or near the output stage using various techniques such as voting, averaging, or weighted aggregation. By decision fusion, the system can benefit from the complementary strengths of different models and improve the accuracy of the final decision. Wang et al. (2022b) designed a best performing system using BERT and RoBERTa feature decision voting with a SVM classifier, regardless of which ASR systems being used, achieving *F*_1_ scores of 93.9% and 91.7%, respectively. Syed et al. (2020) fused the top-10 performing embedding models based on transcripts and achieved an accuracy of 85.4%. Syed et al. (2021) proposed a label fusion system based on deep textual embeddings and LR classifier. By fusing high specificity and high sensitivity models, the paper achieved an accuracy of 84.51%. Qiao et al. (2021) employed model stacking to combine two LRs using complexity and disfluency features respectively, and two models, i.e. BERT and ERNIE, resulting 83.1% accuracy. Wang et al. (2021) fused three CNN-attention networks based on linguistic features and x-vectors using an attention layer followed by a softmax layer, and achieved a good performance. Jin et al. (2023) proposed a complementary and simultaneous ensemble (CONSEN) algorithm to combine the results of prediction and regression tasks, and yielded state-of-the-art performance on the ADReSS-M dataset.

#### 4.3.3. Transfer learning

When it comes to multilingual or low-resource AD detection, transfer learning proves to be a powerful approach for efficiently leveraging patterns from similar tasks and achieving remarkable performance. Recent studies such as Mei et al. (2023), Tamm et al. (2023) have demonstrated the effectiveness of this approach by utilizing pre-training on English datasets and fine-tuning on Greek datasets, resulting in impressive performance for cross-lingual AD detection. This utilization of transfer learning shows its potential in addressing the challenges posed by multilingual and low-resource scenarios in AD detection research.

#### 4.3.4. ASR improvement

Some research tried to improve ASR performance or extract ASR-related features (Pan et al., 2021) for better performance. Ye et al. (2021) exploited a range of techniques to improve ASR performance for older adults to achieve an accuracy of 88%. It is noticed that when using the ground truth transcripts rather than ASR outputs, a comparable or worse performance was obtained with a *F*_1_ score of 87%. Wang et al. (2022a) employed ASR optimization using neural architecture search, cross-domain adaptation and fine-grained elderly speaker adaptation and multi-pass rescoring based system combination with hybrid TDNN.

#### 4.3.5. Combined optimization methods

Many studies have improved the system performance by exploiting more than one kind of optimization methods. For example, Sarawgi et al. (2020) extracted three diverse features and used model fusion strategies, resulting in an accuracy of 88% on Pitt dataset and 83.3% on the ADReSS dataset. Wang et al. (2022a) employed ASR optimization and model fusion strategies based on BERT and RoBERTa features. As a result, the paper achieved state-of-the-art performance with a *F*_1_ score of 92% on the Pitt dataset. Martinc et al. (2021) accounted for temporal aspects of both linguistic and acoustic features by combining ADR with bag-of-n-gram features, and used late fusion via majority vote of 5 classifiers, including Xgboost, RF, SVM, LR and LDA. As a result, the system obtained an appreciable performance with an accuracy of 93.8%.

From Table 2, it is seen that linguistic features extracted from text modality consistently outperform acoustic features extracted from speech. For instance, in the work by Pan et al. (2021), the accuracy of acoustic-only and linguistic-only approaches was reported as 74.65% and 84.51% respectively. Rohanian et al. (2021) revealed that utilizing text modality alone yielded better results than using audio modality, with an accuracy of 74% and 68%, respectively. Then, incorporating diverse features from multiple modalities generally leads to improved performance. For instance, Martinc et al. (2021) demonstrated that the best performance was achieved by combining speech and text modalities, even when text-only features were available. Rohanian et al. (2021) indicated that a multimodal LSTM model with gating outperformed single modality models (0.79 vs. 0.74). Wang et al. (2021) utilized both audio and linguistic features to yield a best performance for AD detection.

Moreover, it is evident that optimization strategies play a crucial role in determining the performance of the studies. Among the various methods employed, model fusion has emerged as an effective approach to achieve better performance in the majority of cases. This demonstrates the significance of optimization strategies and highlights the potential benefits of integrating multiple models for enhanced accuracy and reliability in AD detection studies.

Recently, end-to-end models can directly build a mapping from data to the result label and have achieved promising performance in other fields such as speech processing (Watanabe et al., 2018; He et al., 2019; Yasuda et al., 2021), NLP (Libovickỳ and Helcl, 2018; Xie et al., 2022), CV (Feng et al., 2019; Coquenet et al., 2022). They are also exploited to detect AD recently, such as fine-tuned BERT (Balagopalan et al., 2020), degraded version of generative Transformer (GPT-D) (Li et al., 2022a). However, limited by the size of the publicly available data, the performance of large models does not show significant improvement compared to the utilization of a feature extraction and classification pipeline, with accuracy of 85% lower than the state-of-the-art accuracy of 93.8% (Wang et al., 2022b) on the ADReSS dataset.

## 5. Discussion

ML or DL-based classification models have achieved promising results for the automatic detection of AD. However, there are still some challenges that need to be addressed.

### 5.1. Few-shot and diverse data

There are very few public datasets available until now, with only a limited number of participants, mainly due to the challenges of recording large quantities of audio from AD patients and obtaining expert annotations. Considering the complexity of AD detection, large-scale datasets are necessary for more effective, scalable and powerful models. Moreover, the datasets show a large diversity of accents, languages, neuropsychological tests, background noise and device channels, and thus the best model on one dataset may not have a stable performance on another dataset. Some technologies, such as transfer learning, self-supervised learning or unsupervised learning, data augmentation, provide the potential to address this issue. For example, a recent study by Chen et al. (2023) demonstrated the extraction of paralinguistic features and the feature transfer across English and Greek languages for multilingual AD detection, showing promising results. Additionally, combining an ASR system with speech augmentation and speech enhancement techniques enhances robustness to noise. Beyond the latest studies, more research is required for this challenge.

### 5.2. Model explainability

Although many classification models for AD detection are still based on statistical machine learning algorithm, as shown in Table 2, it can be expected that DL-based methods will be exploited by more studies as the size of the dataset increases in the future because of powerful ability of information representation. However, many of the DL-based models appear as a black box. Thus, it is hard to analyze learned representations and give AD patients any meaningful interpretation, which is often undesirable in medical domain. Some work introduced interpretable NNs to provide interpretation information. For example, Pan et al. (2020) designed SincNet by defining the filters as a collection of parameterized Sinc functions. By analyzing the output of SincNet, a better interpretation of the frequency-related information is gained for cognitive decline assessment. Laguarta and Subirana (2021) introduced the biomarker saliency map to track and visualize progression of AD for the model explainability. However, the explanation provided may not meet the expectations of patients, as they often require a more comprehensive and easily understandable explanation. Additionally, clinicians, who require a deeper and more specialized understanding, also have distinct needs for explanation. Catering to these different requirements for explanation introduces complexity into the model's design and implementation. Recently, there are also many work for interpretable DL used in various fields (Liao et al., 2019; Preuer et al., 2019; Li et al., 2022b), such as drug discovery, glaucoma diagnosis, surveillance of COVID-19, and so on, which can be also used for AD-related tasks to make the model results meaningful.

### 5.3. Model reliability for short recordings

The detection of AD theoretically requires long-term monitoring. However, for most researchers only public datasets are available, and their durations lasting between seconds and minutes. Therefore, the question arises whether such short recordings can provide reliable AD detection results. There is work, such as Laguarta and Subirana (2021), to provide long-term analysis by adding more biomarkers with longitudinal recordings, such as cough. However, the lack of available longitudinal data prevents more researchers from studying this topic.

### 5.4. More modality fusion

This paper reviews automatic detection methods of AD from spontaneous speech, which contains two modalities: audio and text. The use of these two modalities is basically only to extract features separately and then cascade or build separate classification models and fuse them, without aligning the information between modalities. These fusion methods cannot well handle the relationship and interdependence between modalities. Besides, other efficient modalities are also used for AD detection, such as video (MacWhinney et al., 2011), MRI (Chyzhyk et al., 2012; Altinkaya et al., 2020; Noor et al., 2020) and functional MRI images (Wagner, 2000; Ibrahim et al., 2021), video games (Castiblanco et al., 2022), biomakers (Laguarta and Subirana, 2021) and so on. Sheng et al. (2022) fused information from speech and eye-tracking, and achieved a better performance. Pan et al. (2021) designed five models based on BERT, with acoustic features only as model input and combined linguistic features and acoustic features as model input. The detection results showed that the the performance of bimodal-based models outperforms speech only. Future research can use information from more modalities to learn the relationship and interdependence through joint multimodal learning methods.

### 5.5. Distinguishing diseases with similar symptoms

AD is characterized by a range of cognitive and behavioral symptoms, including memory impairment, cognitive decline, emotional and behavioral changes, agitation, aggression, and impairment in daily living activities. These symptoms share similarities with several other medical conditions, which can lead to confusion during early AD diagnosis. For instance, MCI is an early stage of cognitive decline that can be associated with AD but can also occur independently or as a precursor to other types of dementia. Depressive symptoms are also common in AD but can manifest in various other medical conditions as well. Lewy Body Dementia (LBD) is a degenerative brain disorder similar to AD, characterized by the presence of Lewy bodies in brain cells. LBD patients may exhibit AD-like memory problems along with visual hallucinations and motor issues. Thus, Careful differentiation of these similar symptoms is crucial during the early stages of AD diagnosis to establish an accurate assessment. Research by Fraser et al. (2016b) demonstrated the efficiency of MFCC features and SVM classifier in detecting dementia from depression. Pérez-Toro et al. (2023) utilized modified ForestNets to discriminate between AD and depression in AD patients. However, their study did not provide a definitive conclusion regarding the primary distinguishing speech-based symptoms for classifying dementia from other conditions with similar symptoms.

## 6. Conclusions

The paper focuses on the development of automatic AD detection from spontaneous speech, leveraging theoretical basis of the language dysfunction of patients. Compared to other modalities such as MRI, speech-based methods offer non-invasive, convenient and scalable solutions. In this paper, we describe three key components for AD detection in detail, including data collection, feature extraction, and classification models. We also summarize optimization methods and the state-of-the-art performance on several public datasets, with a focus on the last three years. However, AD detection systems face many challenges, and future research can be directed toward improving reliability and accuracy, including increasing dataset sizes or exploring few-shot learning methods, designing interpretable neural networks, establishing long-term monitoring mechanisms (e.g., using wearable devices for real-time monitoring of elderly activities), incorporating multiple modalities and adopting multimodal fusion methods.

The inclusion of cognitive assessments, such as MMSE scores, in longitudinal studies will further advance our understanding of disease progression and its correlation with speech patterns. Future research should consider conducting specialized reviews on AD progression, providing deeper insights into advancements and complementing our current understanding. These efforts will contribute to the development of effective diagnostic tools and treatment strategies for AD.

## Author contributions

Conceptualization: XQ and WB. Methodology, writing—original draft preparation, and project administration: XQ. Investigation: QZ. Writing—review and editing: QZ and JD. Visualization: JD. Supervision: WB. All authors have read and agreed to the published version of the manuscript.
